# Predicting Depression in Older Adults after the COVID-19 Pandemic Using ICF Model

**DOI:** 10.3390/healthcare11081181

**Published:** 2023-04-20

**Authors:** Seonjae Been, Haewon Byeon

**Affiliations:** Department of Digital Anti-Aging Healthcare, Graduate School (BK21), Inje University, Gimhae 50834, Republic of Korea

**Keywords:** COVID-19, pandemic, older adults, depression, International Classification of Functioning, Disability, and Health

## Abstract

This study aimed to test a predictive model for depression in older adults in the community after the COVID-19 pandemic and identify influencing factors using the International Classification of Functioning, Disability, and Health (ICF). The subjects of this study were 9920 older adults in South Korean local communities. The analysis results of path analysis and bootstrapping analysis revealed that subjective health status, instrumental activities of daily living (IADL), number of chronic diseases, social support satisfaction, household economic level, informal support, and participation in social groups were factors directly influencing depression, while formal support, age, gender, education level, employment status, and participation in social groups were factors indirectly affecting it. It will be needed to prepare measures to prevent depression in older adults during an infectious disease pandemic, such as the COVID-19 pandemic, based on the results of this study.

## 1. Introduction

As COVID-19 rapidly spread after it entered South Korea in January 2020, social distancing, a quarantine policy, was implemented. Non-face-to-face and non-contact type social distancing caused socio-environmental changes (e.g., changes in the work environment [1,2], an increase in leisure time spent alone [1,2], and the acceleration of digital transformation) and negative changes (e.g., reduced daily life and decreased frequency in leisure activities and club participation due to anxiety and fear, and social isolation, loneliness, and stress because of self-isolation) at the same time [3,4,5]. The spread of the COVID-19 virus had such a negative impact on mental health it created a new term, “corona blues”, which referred to depression and lethargy induced by COVID-19 [6].

These social, physical, and mental problems decreased the amount of exercise and nutritional intake and suspended economic activities to affect the lives of older adults more diversely and complexly [7]. The increased risk of infection could have impacted the isolation of older adults from society and, as a result, their mental health. Especially, restrictions on outdoor activities isolated older adults from society, which disconnected their social networks. When older adults’ social networks are weakened and broken, they may suffer from loneliness, insomnia, and stress, which can increase the frequency of feeling depressed [8,9,10]. Moreover, recent studies showed that the risk of developing depression in older adults doubled after the COVID-19 pandemic, and older adults who had not been exposed to depression in the past also had a 2.4-fold increased risk of developing depression [11].

Depression has long been recognized as a biopsychosocial model that involves the interaction of biological, psychological, and social factors. Various socio-economic, physical, and psychological factors such as gender, income level, physical function, and anxiety have been found to influence depression even before the COVID-19 pandemic [12,13,14].

As the COVID-19 pandemic has brought changes in various sectors, it is necessary to explore them accordingly. Therefore, this study used the International Classification of Functioning, Disability, and Health (ICF) to systematically classify depression in older adults in the community from various perspectives.

The components of the ICF are divided into six categories: health conditions such as disabilities and diseases, body functions and structures, activities, participation, environmental factors, and personal factors. It is explained that these components have complex relationships or interactions. Therefore, ICF can be used to explore the association and causal relationship between each component using collected individual data, and it is useful when comprehensively explaining the experience of health status [15].

**Figure 1:**
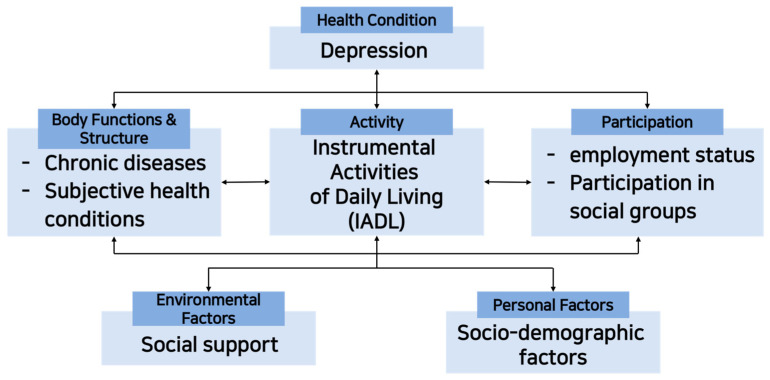
Composition and variables of the International Classification of Functioning, Disability, and Health.

Since older adults were suspected of being at high risk for depression due to the COVID-19 pandemic, this study aimed to construct and test a predictive model that could explain depression in older adults living in the community based on the ICF components (Figure 1). In addition, this study applied the ICF model based on the factors reported to affect depression in the elderly in previous review studies [16,17,18], (1) to identify the relationship between the factors affecting depression in the elderly, and (2) analyze how each factor affects depression.

## 2. Materials and Methods

### 2.1. Research Model and Data Source

#### 2.1.1. Model Framework

**Figure 2:**
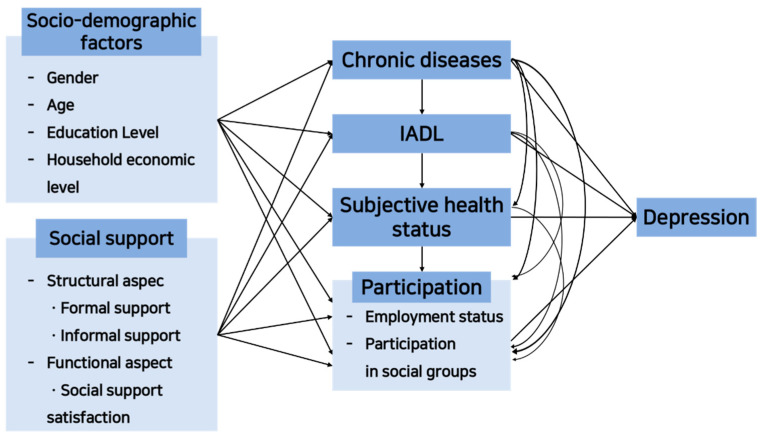
Model framework.

This study established a model framework (Figure 2) to identify the structural relationships between variables affecting depression in older adults living in the community and explain the relationship between variables after the COVID-19 pandemic based on the ICF model.

#### 2.1.2. Data Source

This study utilized data from the 2020 Survey of the Living Condition of the Elderly conducted by the Ministry of Health and Welfare and the Korea Institute for Health and Social Affairs. The Survey of the Living Condition of the Elderly aims to produce baseline data and basic indicators necessary for establishing policies for older adults in accordance with the rapid increase in the older adult population. It is conducted every three years because the implementation of the Survey of the Living Condition of the Elderly was enacted under the Welfare of the Aged Act. The target population of the 2020 Survey of the Living Condition of the Elderly was older adults (≥65 years old) residing in general residential facilities in 17 South Korean cities and provinces. Samples were extracted using the stratified two-stage cluster sampling method after stratifying the samples by 17 cities and provinces and dong/eup/myeon. Data collection was conducted from 14 September to 20 November 2020, and pre-trained surveyors visited sample households and conducted 1:1 face-to-face interviews using a standardized questionnaire [19]. This study analyzed the data of 9920 people, after excluding older adults living alone whose social support could not be measured because they did not have surviving children or grandchildren, taken from among 10,097 older adults (≥65 years old) who participated in the 2020 Survey of the Living Condition of Elderly.

### 2.2. Measurement and Definition of Variables

#### 2.2.1. Depression

Depression is the outcome variable, and it was defined by the Geriatric Depression Scale Short Form-Korea version (GDS-SF-K) [20], which was the standardized Geriatric Depression Scale (GDS), developed by Sheikh and Yesavage, according to the characteristics of Korean older adults. The GDS-SF-K consists of 15 items, and respondents should respond in a binary format (yes or no) based on their experiences within the past week. The score ranges from 0 to 15, and this study defined the cut-off score as 8 points by referring to Lee et al. [21]. The reliability of the GDS-SF-K (Cronbach’s α) was 0.88 [20], and its reliability in this study (Cronbach’s α) was 0.90.

#### 2.2.2. Personal Factors: Socio-Demographic Factors

Personal factors included gender (male or female), age (65–74, 75–84, 85 and older), education level (elementary school graduation or below, middle school graduation, high school graduation, or college graduation or above), and household economic level (national basic livelihood security recipients or national basic livelihood security non-recipients).

#### 2.2.3. Environmental Factors: Social Support

Social support was defined by dividing it into the structural aspect (i.e., informal support and formal support) and the functional aspect (i.e., social support satisfaction). The informal support of the structural aspect was measured by using the frequency (I met an acquaintance two times or fewer in a year, I met an acquaintance 1–2 times every 3 months, I met an acquaintance one or two times a month, I met an acquaintance once a week, or I met an acquaintance almost every day) of meeting acquaintances, e.g., non-live-in children (including children’s spouses), grandchildren, brothers, sisters, relatives, friends, or neighbors in the past year. The formal support of the structural aspect was measured by utilizing a senior citizen center or senior welfare service center (I used it, or I did not use it) in the community in the past year. The social support satisfaction of the functional aspect was measured using relationship satisfaction with children (satisfied, not satisfied, or dissatisfied) and relationship satisfaction with friends/neighbors (satisfied, not satisfied, or dissatisfied).

#### 2.2.4. Body Functions and Structures: Chronic Diseases and Subjective Health Conditions

Chronic disease was defined as the number of chronic diseases diagnosed by a doctor and suffered for 3 months or longer (≤1, 2–4, or ≥5) among 31 chronic diseases, such as hypertension, diabetes, urinary incontinence, presbycusis, and cataracts. Subjective health status was defined as “poor, average, or good”.

#### 2.2.5. Activities: Instrumental Activities of Daily Living (IADL)

The Instrumental Activities of Daily Living (IADL) were measured using the Korea Instrumental Activities of Daily Living Scale (K-IADL) [22]. The K-IADL consists of 10 items, and respondents respond based on the activities in the past week. Seven items (dressing, housework, meal preparation, laundry, taking medicine, money management, and short distance outing) were measured using a 3-point scale (completely independent = 1, partially independent = 2, or completely dependent = 3). Shopping for goods, using the phone, and using transportation were measured on a 4-point scale (completely independent = 1, requiring little help = 2, requiring a lot of help = 3, or completely dependent = 4). It was recategorized into completely independent (1), partially independent (2), or completely dependent (3). All measurements were reversely coded to make a higher score indicating higher independence. This study defined people who obtained 25 points or lower as a group with difficulty in IADL and those who obtained 26 points or higher as a group without difficulty in IADL. At the time of development, the reliability of the tool (Cronbach’s α) was 0.94, and that of the previous studies (Cronbach’s α) was 0.95, which were high [23]. In this study, Cronbach’s α was 0.88.

#### 2.2.6. Social Participation: Employment Status and Participation in Social Groups

Social participation was defined as employment status (no or yes) and participation in social groups (no or yes).

### 2.3. Analysis Methods

This study analyzed the collected data using SPSS 27 and Amos 27.0. The general characteristics of the subjects were analyzed by frequency analysis or descriptive statistics. The model was verified using path analysis, and the goodness of fit criteria of the model were CMIN/DF < 3, GFI ≥ 0.90, AGFI ≥ 0.85, CFI ≥ 0.95, TLI ≥ 0.95, RMSEA ≤ 0.06, and SRMR ≤ 0.08 [24]. The significance of the path was examined by using path coefficient estimates and critical ratios (C.R.). Parameter estimates were decomposed into direct and indirect effects, and the statistical significance of the total and indirect effects was tested using bootstrapping analysis.

## 3. Results

### 3.1. General Characteristics

Among the total study population of 9920 individuals, 8627 (87%) did not have depression, while 1293 (13%) had depression. Table 1 displays the general characteristics of the elderly community according to the prevalence of depression.

### 3.2. Predictive Model Verification

#### 3.2.1. Verification of Predictive Model’s Goodness of Fit

Table 2 shows the results of the goodness of fit verification of the predictive model. The results revealed that the predictive model’s goodness of fit was good (CMIN/DF = 1.653, GFI = 1.000, AGFI = 0.998, SRMR = 0.0037, TLI = 0.997, CFI = 0.999, and RMSEA = 0.008).

#### 3.2.2. Estimation of Predictive Model’s Path Coefficients

**Figure 3:**
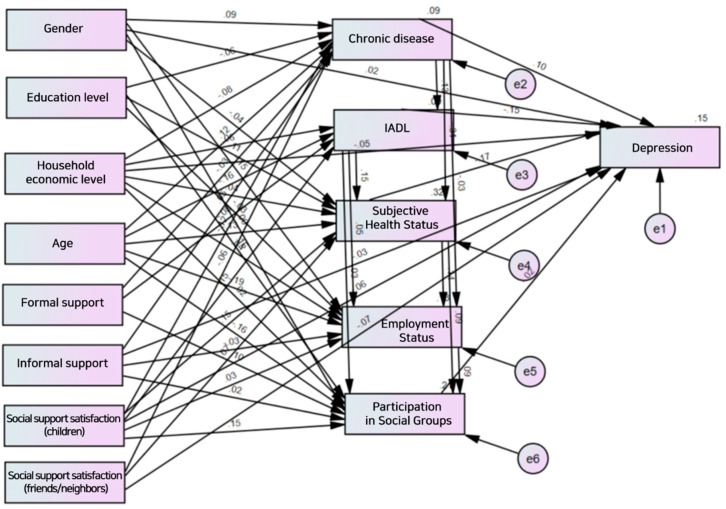
Standardization coefficients of the predictive model.

Table 3 and Figure 3 show the analysis results of the predictive model. The results showed that the subject had more chronic diseases when they were females (β = 0.092, *p* < 0.001), had lower education levels (β = −0.063, *p* < 0.001), received national basic livelihood security (β = −0.081, *p* < 0.001), was older (β = 0.118, *p* < 0.001), had higher informal support (β = 0.049, *p* < 0.001), had lower formal support (β = −0.029, *p* < 0.01), and had lower social support satisfaction (friends/neighbors, and children), which was functional support referring to the satisfaction with a specific relationship (β = −0.117, *p* < 0.001, and β = −0.061, *p* < 0.001, respectively). These variables explained 8.8% of the variance.

The IADL was higher when the subject did not receive national basic livelihood security (β = 0.061, *p* < 0.001), was younger (β = −0.162, *p* < 0.001), had lower formal support (β = −0.079, *p* < 0.001), had higher relationship satisfaction with friends/neighbors (β = 0.148, *p* < 0.001), and had fewer chronic diseases (β = −0.130, *p* < 0.001). These variables explained 9.5% of the variance.

Subjective health status was better when the subject was male (β = −0.039, *p* < 0.001), had a higher education level (β = 0.112, *p* < 0.001), did not receive national basic livelihood security (β = 0.040, *p* < 0.001), was younger (β = −0.134, *p* < 0.001), had higher social support satisfaction (friends/neighbors, and children) (β = 0.151, *p* < 0.001, and β = 0.067, *p* < 0.001, respectively), had fewer chronic diseases (β = −313, *p* < 0.001), and had higher IADL scores (β = 0.153, *p* < 0.001). These variables explained 32.3% of the variance.

The subject was more likely to be employed when the subject was male (β = −0.152, *p* < 0.001), had a lower education level (β = −0.025, *p* < 0.05), did not receive national basic livelihood security (β = 0.038, *p* < 0.001), was younger (β = −0.189, *p* < 0.001), had lower informal support (β = −0.032, *p* < 0.001), had higher relational satisfaction with friends/neighbors (β = 0.032, *p* < 0.01), had fewer chronic diseases (β = −0.03, *p* < 0.01), had higher IADL scores (β = 0.046, *p* < 0.001), and had better subjective health status (β = 141, *p* < 0.001). These variables explained 12.0% of the variance.

The subject was more likely to participate in social groups when the subject had a higher education level (β = 0.163, *p* < 0.001), was younger (β = −0.162, *p* < 0.001), had higher formal support (β = 0.096, *p* < 0.001), had lower informal support (β = 0.022, *p* < 0.05), had higher satisfaction in a relationship with friends/neighbors (β = 0.152, *p* < 0.001), had higher IADL scores (β = 0.027, *p* < 0.01), had better subjective health status (β = 0.091, *p* < 0.001), and was employed (β = 0.092, *p* < 0.001). These variables explained 21.4% of the variance.

It was found that the subject was more likely to suffer from depression when the subject received national basic livelihood security (β = −0.052, *p* < 0.001), had lower informal support (β = −0.031, *p* < 0.001), had lower social support satisfaction (friends/neighbors, and children) (β = −0.065, *p* < 0.001, and β = −0.075, *p* < 0.001, respectively), had more chronic diseases (β = 0.1, *p* < 0.001), had lower IADL scores (β = −0.149, *p* < 0.001), had poorer subjective health status (β = −0.168, *p* < 0.001), and did not participate in social groups (β = −0.021, *p* < 0.05). These variables explained 14.7% of the variance.

#### 3.2.3. Effect Analysis of the Predictive Model

Table 4 shows the total effect, direct effect, and indirect effect of this study. It was confirmed that social support satisfaction (children and friends/neighbors), formal support, informal support, age, household economic level, education level, and gender had significant direct effects on chronic disease.

Variables affecting IADL were social support satisfaction (children and friends/neighbors), formal support, informal support, age, household economic level, education level, gender, and chronic disease. Among them, the total effect, direct effect, and indirect effect of relationship satisfaction with friends/neighbors, formal support, age, and household economic level were significant. Moreover, the total and indirect effects of relationship satisfaction with children, informal support, and gender were significant. It was also confirmed that only the indirect effect of education level and the direct effect of chronic disease were significant.

It was confirmed that social support satisfaction (children and friends/neighbors), informal support, age, household economic level, education level, gender, number of chronic diseases, and IADL were variables affecting subjective health status. Among them, the total effect, direct effect, and indirect effect of social support satisfaction (children and friends/neighbors), age, household economic level, education level, gender, and chronic disease were significant. The total effect and indirect of informal support were significant, while the indirect effect of IADL was significant.

Variables affecting employment status were social support satisfaction (children and friends/neighbors), formal support, informal support, age, household economic level, education level, gender, chronic disease, IADL, and subjective health status. The total effect, direct effect, and indirect effect of relationship satisfaction with friends/neighbors, informal support, age, household economic level, education level, gender, number of chronic diseases, and IADL were significant. The total effect and indirect effect of relationship satisfaction with children and formal support were significant, while the direct effect of subjective health status was significant.

Social support satisfaction (children and friends/neighbors), formal support, informal support, age, household economic level, education level, gender, chronic disease, IADL, subjective health status, and employment status influenced social group participation. The total effect, direct effect, and indirect effect of relationship satisfaction with friends/neighbors, formal support, informal support, age, IADL, and subjective health status were significant. The total and indirect effects of relationship satisfaction with children, household economic level, gender, and chronic disease were significant, while the direct and indirect effects of education level were significant. In addition, it was found that the direct effect of employment status was significant.

It was confirmed that social support satisfaction (children and friends/neighbors), formal support, informal support, age, household economic level, education level, gender, chronic disease, IADL, subjective health status, employment status, and participation in social groups were factors affecting depression. The total effect, direct effect, and indirect effect of social support satisfaction (children and friends/neighbors), informal support, household economic level, chronic disease, IADL, and subjective health status were significant. The total effect and indirect effect of formal support, age, education level, gender, and employment status were significant, while the indirect effect of participation in social groups was significant.

## 4. Discussion

This study evaluated the predictive model of variables influencing depression in older adults living in the community after the COVID-19 pandemic based on the ICF model and analyzed the effects of each variable. The results showed that subjective health status directly and negatively affected depression (β = −0.168, *p* < 0.001), influencing depression the most among ICF factors. The results indicated that people who evaluated their subjective health status as poorer were more likely to have depression. Therefore, based on this study, continuous mental health monitoring at the community level is needed to prevent depression in the elderly, especially those reporting poor subjective health during infectious diseases like the COVID-19 pandemic.

The study also identified the number of chronic diseases and instrumental activities of daily living (IADL) as the variables that affect subjective health status. The results were similar to those of previous studies that analyzed factors related to depression in older adults [25,26,27]. The results of previous studies suggested that it would be necessary to manage chronic diseases and improve IADL to enhance subjective health status [28,29]. Consequently, improved physical ability and exercise are needed to achieve these goals [28,29]. Therefore, it would be necessary to develop a fellowship program for enhancing physical abilities, in addition to mental health, for older adults in the community.

We found that relationship satisfaction with friends and neighbors, as well as informal support, impacted chronic disease, IADL, subjective health status, employment status, social group participation, and depression. Our study suggests that non-family individuals, such as friends, neighbors, and acquaintances, impact various aspects of the elderly’s lives.

Lastly, the results of this study showed that gender did not have a significant direct impact on depression, which disagreed with the results of previous studies showing that gender influenced depression in older adults [30,31,32,33]. The discrepancy could be because this study evaluated depression during the COVID-19 pandemic, while the previous studies were conducted before the COVID-19 pandemic. It is assumed that gender had little direct effect on depression during the COVID-19 pandemic. Although many studies have evaluated depression and the factors associated with it, additional studies are needed to prove the true relationship because not many studies examined depression during the COVID-19 pandemic, a special period.

This study’s strength lies in its comprehensive exploration of the factors that affect depression in the elderly after the COVID-19 pandemic, using a sample representative of the elderly in the community. Our study also identified the relationship between variables applying the ICF model. The limitations of this study are as follows. First, this study did not measure the frequency of participation in social groups among the social participation used in this study. Second, this was a cross-sectional study, and it tested the hypothetical causal relationship using a predictive model. It will be necessary to conduct longitudinal studies to identify a precise causal of variables on depression. Third, the data used in this study did not contain information regarding the time of depression onset. Therefore, future studies need to examine the relationship after including the time of depression onset using additional medical records. Forth, a study with a larger sample size that considers the ratio of subjects with IADL difficulties and those without IADL difficulties is needed, as the data used in our study had a low percentage of subjects with IADL difficulties.

## 5. Conclusions

This study identified factors influencing depression in older adults in the community after the COVID-19 pandemic using ICF components. The results showed that subjective health status, IADL, number of chronic diseases, social support satisfaction, household economic level, informal support, and participation in social groups, in order of magnitude, had a direct impact on depression, while formal support, age, gender, education level, employment status, and participation in social groups had an indirect effect. Future longitudinal studies or community-based cohort studies are needed to confirm the causal relationship between depression in older adults and associated variables under special circumstances, such as an infectious disease pandemic period, along with considering medical records.

## Figures and Tables

**Table 1:** General characteristics of study subjects.

Subgroups	Classification	Variables		*N* (%)
No Depression	Personal Factors	Gender	Male	3549 (41.1)
			Female	5078 (58.9)
		Age	65–74 years old	5367 (62.2)
			75–84 years old	2782 (32.2)
			≥85 years old	478 (5.5)
		Education Level	Elementary school graduation or below	3665 (42.5)
			Middle school graduation	2028 (23.5)
			High school graduation	2468 (28.6)
			College graduation or above	466 (5.4)
		Household economic level	National basic livelihood security recipient	519 (6.0)
			national basic livelihood security non-recipient	8108 (94.0)
	Environmental Factors	Formal support (used a senior citizen center or senior welfare service center in the community in the past year)	Used	2557 (29.6)
			Never used	6070 (70.4)
		Informal support (frequency of meeting an acquaintance in the past year)	≥2 times a year	1381 (16.0)
			1–2 times every 3 months	2464 (28.6)
			1–2 times a month	3371 (39.1)
			Once a week	908 (10.5)
			Everyday	503 (5.8)
		Social support satisfaction (relationship with children)	Dissatisfied	236 (2.7)
			Not satisfied or dissatisfied	1729 (20.2)
			Satisfied	6662 (77.2)
		Social support satisfaction (relationship with friends/neighbors)	Dissatisfied	359 (4.2)
			Not satisfied or dissatisfied	2867 (33.2)
			Satisfied	5401 (62.6)
	Physical Functions and Structures	Chronic disease	≤1	4286 (49.7)
			2–4	4082 (47.3)
			≥5	259 (3.0)
		Subjective health status	Poor	1216 (14.1)
			Average	2740 (31.8)
			Good	4671 (54.1)
	Activities	Instrumental Activities of Daily Living (IADL)	≤25 points	190 (2.2)
			≥26 points	8437 (97.8)
	Social Participation	Employment status	No	5304 (61.5)
			Yes	3323 (38.5)
		Participation in social groups	No	4806 (55.7)
			Yes	3821 (44.3)
Depression	Personal Factors	Gender	Male	422 (32.6)
			Female	871 (67.4)
		Age	65–74 years old	610 (47.2)
			75–84 years old	551 (42.6)
			≥85 years old	132 (10.2)
		Education Level	Elementary school graduation or below	766 (59.2)
			Middle school graduation	302 (23.4)
			High school graduation	186 (14.4)
			College graduation or above	39 (3.0)
		Household economic level	National basic livelihood security recipient	206 (15.9)
			national basic livelihood security non-recipient	1087 (84.1)
	Environmental Factors	Formal support (used a senior citizen center or senior welfare service center in the community in the past year)	Used	401 (31.0)
			Never used	892 (69.0)
		Informal support (frequency of meeting an acquaintance in the past year)	≥2 times a year	305 (23.6)
			1–2 times every 3 months	334 (25.8)
			1–2 times a month	458 (35.4)
			Once a week	126 (9.7)
			Everyday	70 (5.4)
		Social support satisfaction (relationship with children)	Dissatisfied	145 (11.2)
			Not satisfied or dissatisfied	387 (29.9)
			Satisfied	761 (58.9)
		Social support satisfaction (relationship with friends/neighbors)	Dissatisfied	253 (19.6)
			Not satisfied or dissatisfied	513 (39.7)
			Satisfied	527 (40.8)
	Physical Functions and Structures	Chronic disease	≤1	314 (24.3)
			2–4	754 (58.3)
			≥5	225 (17.4)
		Subjective health status	Poor	644 (49.8)
			Average	380 (29.4)
			Good	269 (20.8)
	Activities	Instrumental Activities of Daily Living (IADL)	≤25 points	214 (16.6)
			≥26 points	1079 (83.4)
	Social Participation	Employment status	No	1002 (77.5)
			Yes	291 (22.5)
		Participation in social groups	No	973 (75.3)
			Yes	320 (24.7)

**Table 2:** The goodness of fit of the predictive model.

CMIN/DF	GFI	AGFI	SRMR	TLI	CFI	RMSEA
1.653	1.000	0.998	0.0037	0.997	0.999	0.008

**Table 3:** Standardized coefficient and squared multiple correlations (SMC) of the predictive model.

Path		β	S.E.	C.R.	SMC
Chronic Disease	← Gender	0.092	0.012	9.114 ***	0.088
	← Education level	−0.063	0.007	−5.623 ***	
	← Household economic level	−0.081	0.022	−8.245 ***	
	← Age	0.118	0.010	11.034 ***	
	← Informal support	0.049	0.005	5.11 ***	
	← Formal support	−0.029	0.013	−2.799 **	
	← Social support satisfaction (friends/neighbors)	−0.117	0.010	−11.078 ***	
	← Social support satisfaction (children)	−0.061	0.011	−5.814 ***	
IADL	← Household economic level	0.061	0.007	6.228 ***	0.095
	← Age	−0.162	0.003	−15.796 ***	
	← Formal support	−0.079	0.004	−7.902 ***	
	← Social support satisfaction (friends/neighbors)	0.148	0.003	14.992 ***	
	← Chronic disease	−0.130	0.003	−13.116 ***	
Subjective Health Status	← Gender	−0.039	0.014	−4.465 ***	0.323
	← Education level	0.112	0.008	11.741 ***	
	← Household economic level	0.040	0.025	4.696 ***	
	← Age	−0.134	0.012	−14.644 ***	
	← Social support satisfaction (friends/neighbors)	0.151	0.012	16.422 ***	
	← Social support satisfaction (children)	0.067	0.013	7.542 ***	
	← Chronic disease	−0.313	0.011	−35.892 ***	
	← IADL	0.153	0.034	17.707 ***	
Employment Status	← Gender	−0.152	0.010	−15.319 ***	0.128
	← Education level	−0.025	0.006	−2.296 *	
	← Household economic level	0.038	0.018	3.944 ***	
	← Age	−0.189	0.008	−17.927 ***	
	← Informal support	−0.032	0.004	−3.408 ***	
	← Social support satisfaction (friends/neighbors)	0.032	0.008	3.229 **	
	← Chronic disease	−0.030	0.009	−2.816 **	
	← IADL	0.046	0.024	4.613 ***	
	← Subjective health status	0.141	0.007	12.436 ***	
Participation in Social Groups	← Education level	0.163	0.005	15.469 ***	0.214
	← Household economic level	0.016	0.017	1.765	
	← Age	−0.162	0.008	−15.648 ***	
	← Formal support	0.096	0.010	10.06 ***	
	← Informal support	0.022	0.004	2.456 *	
	← Social support satisfaction (friends/neighbors)	0.152	0.008	15.791 ***	
	← Gender	0.001	0.010	0.099	
	← IADL	0.027	0.024	2.797 **	
	← Subjective health status	0.091	0.007	8.878 ***	
	← Employment status	0.092	0.010	9.658 ***	
Depression	← Gender	0.015	0.006	1.631	0.147
	← Household economic level	−0.052	0.012	−5.433 ***	
	← Informal support	−0.031	0.003	−3.298 ***	
	← Social support satisfaction (friends/neighbors)	−0.065	0.006	−6.156 ***	
	← Social support satisfaction (children)	−0.075	0.006	−7.427 ***	
	← Chronic disease	0.100	0.006	9.591 ***	
	← IADL	−0.149	0.017	−15.12 ***	
	← Subjective health status	−0.166	0.005	−14.893 ***	
	← Participation in social groups	−0.021	0.007	−2.138 *	

* *p* < 0.05, ** *p* < 0.01, *** *p* < 0.001.

**Table 4:** Effect analysis of the predictive model.

Path		Total Effect	Direct Effect	Indirect Effect
Chronic Disease	← Social support satisfaction (children)	−0.061 ***	−0.061 ***	-
	← Social support satisfaction (friends/neighbors)	−0.117 ***	−0.117 ***	-
	← Formal support	−0.029 ***	−0.029 **	-
	← Informal support	0.049 ***	0.049 ***	-
	← Age	0.118 ***	0.118 ***	-
	← Household economic level	−0.081 ***	−0.081 ***	-
	← Education level	−0.063 ***	−0.063 ***	-
	← Gender	0.092 ***	0.092 ***	-
IADL	← Social support satisfaction (children)	0.008 **	0	0.008 ***
	← Social support satisfaction (friends/neighbors)	0.164 ***	0.148 ***	0.015 ***
	← Formal support	−0.076 ***	−0.079 ***	0.004 **
	← Informal support	−0.006 ***	0	−0.006 ***
	← Age	−0.177 ***	−0.162 ***	−0.015 ***
	← Household economic level	0.071 ***	0.061 ***	0.010 ***
	← Education level	0.008	0	0.008 ***
	← Gender	−0.012 ***	0	−0.012 ***
	← Chronic disease	−0.130 ***	−0.130 ***	-
Subjective Health Status	← Social support satisfaction (children)	0.087 ***	0.067 ***	0.020 ***
	← Social support satisfaction (friends/neighbors)	0.212 ***	0.151 ***	0.062 ***
	← Formal support	−0.003	0	−0.003
	← Informal support	−0.016 ***	0	−0.016 ***
	← Age	−0.199 ***	−0.134 ***	−0.064 ***
	← Household economic level	0.076 ***	0.040 ***	0.036 ***
	← Education level	0.133 ***	0.112 ***	0.021 ***
	← Gender	−0.070 ***	−0.039 ***	−0.031 ***
	← Chronic disease	−0.332 ***	−0.313 ***	−0.020 ***
	← IADL	0.153 ***	0.153 ***	-
Employment Status	← Social support satisfaction (children)	0.015 ***	0	0.015 ***
	← Social support satisfaction (friends/neighbors)	0.073 ***	0.032 ***	0.041 ***
	← Formal support	−0.003 **	0	−0.003 *
	← Informal support	−0.036 ***	−0.032 **	−0.004 ***
	← Age	−0.228 ***	−0.189 ***	−0.040 ***
	← Household economic level	0.054 ***	0.038 ***	0.016 ***
	← Education level	−0.004 ***	−0.025 **	0.021 ***
	← Gender	−0.165 ***	−0.152 ***	−0.013 ***
	← Chronic disease	−0.083 ***	−0.030 **	−0.053 ***
	← IADL	0.068 ***	0.046 ***	0.022 ***
	← Subjective health status	0.141 ***	0.141 ***	-
Participation in Social Groups	← Social support satisfaction (children)	0.009 ***	0	0.009 ***
	← Social support satisfaction (friends/neighbors)	0.182 ***	0.152 ***	0.030 ***
	← Formal support	0.094 ***	0.096 ***	−0.003 **
	← Informal support	0.017 *	0.022 **	−0.005 ***
	← Age	−0.206 ***	−0.162 ***	−0.044 ***
	← Household economic level	0.030 ***	0.016	0.014 ***
	← Education level	0.175	0.163 ***	0.012 ***
	← Gender	−0.021 *	0.001	−0.022 ***
	← Chronic disease	−0.041 ***	0	−0.041 ***
	← IADL	0.047 ***	0.027 ***	0.020 ***
	← Subjective health status	0.104 ***	0.091 ***	0.013 ***
	← Employment status	0.092 ***	0.092 ***	-
Depression	← Social support satisfaction (children)	−0.097 ***	−0.075 ***	−0.022 ***
	← Social support satisfaction (friends/neighbors)	−0.140 ***	−0.065 ***	−0.075 ***
	← Formal support	0.007 *	0	0.007 *
	← Informal support	−0.023 *	−0.031 ***	0.008 ***
	← Age	0.075 ***	0	0.075 ***
	← Household economic level	−0.083 ***	−0.052 ***	−0.032 ***
	← Education level	−0.033 ***	0	−0.033 ***
	← Gender	0.038 ***	0.015	0.023 ***
	← Chronic disease	0.175 ***	0.100 ***	0.075 ***
	← IADL	−0.175 ***	−0.149 ***	−0.026 ***
	← Subjective health status	−0.168 ***	−0.166 ***	−0.002 *
	← Employment status	−0.002 *	0	−0.002 *
	← Participation in social groups	−0.021 *	−0.021 *	-

* *p* < 0.05, ** *p* < 0.01, *** *p* < 0.001.

## Data Availability

The data presented in this study are provided at the request of the corresponding author. The data is not publicly available because researchers need to obtain permission from the Korea Centers for Disease Control and Prevention.
